# Optimization of Chromosome Preparation and Karyotype Analysis of Winter Turnip Rape (*Brassica rape* L.)

**DOI:** 10.3390/ijms26157127

**Published:** 2025-07-24

**Authors:** Tingting Fan, Xiucun Zeng, Yaozhao Xu, Fei Zhang, Li Ma, Yuanyuan Pu, Lijun Liu, Wangtian Wang, Junyan Wu, Wancang Sun, Gang Yang

**Affiliations:** 1 College of Agronomy, Gansu Agricultural University, Lanzhou 730070, China; fantt@gsau.edu.cn (T.F.);; 2 State Key Laboratory of Arid Land Crop Science, Lanzhou 730070, China; 3 College of Life Science and Engineering, Hexi University, Zhangye 734000, China; 4 Key Laboratory of Hexi Corridor Resources Utilization of Gansu, Hexi University, Zhangye 734000, China; 5 College of Agriculture and Ecological Engineering, Hexi University, Zhangye 734000, China; 6 College of Life Science and Technology, Gansu Agricultural University, Lanzhou 730070, China

**Keywords:** Longyou 7, chromosome preparation, karyotype formula, phylogenetic evolution

## Abstract

To explore the dyeing technique and karyotype analysis of winter turnip rape (*Brassica rape* L.), the root tip of winter turnip rape Longyou 7 was used as the experimental material. Chromosome preparation technology was optimized, and karyotype analysis was carried out by changing the conditions of material collection time, pretreatment, fixation, and dissociation. The results showed that the optimal conditions for the preparation of dyeing winter turnip rape were as follows: the sampling time was 8:00–10:00, the ice–water mixture was pretreated at 4 °C for 20 h, the Carnot’s fixative solution I and 4 °C were fixed for 12 h, and the 1 mol/L HCl solution was bathed in a water bath at 60 °C for 10~15 min. Karyotype analysis showed that the number of chromosomes in winter turnip rape cells was 2n = 20, and the karyotype analysis formula was 2n = 2x = 20 = 16m + 4sm. The karyotype asymmetry coefficient was 58.85%, and the karyotype type belonged to type 2A, which may belong to the primitive type in terms of evolution. The results of this study provide a theoretical basis for further in-depth study of the phylogenetic evolution and genetic trend of *Brassica rapa*.

## 1. Introduction

Winter turnip rape evolved from *Brassica campestris* L. It has a short plant stature, with smaller branches and slender stems. It has thin and smooth oval leaves. There are obvious lyrate incisions on the leaf margins with prickles on the leaves, and it is covered with a thin layer of wax powder. It is also known as mini rape, dwarf rape, and sweet rape. In northern China, the winter temperature can drop as low as minus 32 °C, and drought often occurs in spring. The ecological conditions are extremely harsh [1,2]. The problems of wind erosion and farmland degradation are severe, and the desertified areas are constantly expanding. As the only winter oilseed crop in northern China, the successful northward transplantation of winter rapeseed has brought economic benefits to the northern regions. In addition, winter rapeseed is also used as a winter cover crop. It can effectively increase the vegetation coverage rate in winter and spring, improve the multiple cropping index and land utilization rate, reduce soil wind erosion, increase the content of soil organic matter, protect farmland, promote the balanced development of local economic production, and achieve ecological and environmental protection [3]. At present, the research on winter turnip rape mainly centers on its cold-resistance mechanism [3,4,5,6,7,8]. In contrast, there are few cytological reports regarding its plant chromosome preparation and karyotype parameters.

Karyotype analysis, as the core technique for the qualitative and quantitative evaluation of plant chromosome characteristics, plays an irreplaceable role in analyzing genome structural variations, tracing phylogenetic relationships, and guiding genetic improvement practices. In studies on polyploidization origins and species evolution, karyotype analysis has proven to be a critical breakthrough point [9]. Through the study of characteristics such as chromosome length, arm ratio, and the position of the centromere, karyotype analysis can provide crucial evidence for plant taxonomy. Especially when resolving taxonomic disputes, karyotype data can serve as important reference evidence [10]. Through the karyotype analysis of the apomictic plant *Boechera*, scholars have discovered the origin of abnormal chromosomes, further confirming the status of cruciferous plants as an important model system for studying the evolution of plant chromosomes and genomes [11]. Additionally, karyotype analysis can reveal the role of polyploidization in plant evolution, such as showing chromosome number variations among different *Gazania rigens* varieties. It was speculated that the basic chromosome number was x = 5, and some varieties might be tetraploid. This provided important clues for understanding the origin and evolution of the species [12]. A classic example is the *Coffea* karyotype identification, which revealed *Coffea arabica* as an allotetraploid (2n = 44), while the majority of its closely related species are diploids (2n = 22). This directly confirms its evolutionary pathway formed through interspecific hybridization followed by genome doubling [9]. Similarly, the identification of ploidy characteristics in germplasms such as Guizhou cherry is of fundamental significance for clarifying the genetic relationships among germplasm resources, optimizing the utilization of regional genetic resources, and designing precise breeding strategies [13].

At the level of chromosome structure and environmental adaptability, quantitative differences in karyotype parameters can reflect the shaping influence of ecological pressures on the genome. For instance, distinct geographical populations of the common bean (*Phaseolus vulgaris*) exhibit significant karyotypic differentiation. Karyotype asymmetry indices, comprising variations in chromosome length (interchromosomal asymmetry) and changes in the centromeric index (intrachromosomal asymmetry), have become key indicators for quantifying a species’ evolutionary status and adaptive potential to its environment [14]. In the realms of germplasm resource development and taxonomic applications, karyotype analysis demonstrates dual value. On one hand, comparative studies of karyotypic diversity, such as those conducted on six cultivated varieties and nine wild relatives (including diploids and tetraploids) of Indian rice, elucidate interspecific differentiation and intraspecific evolutionary relationships. On the other hand, germplasm evaluation based on karyotypic features (e.g., ploidy, asymmetry coefficients) provides theoretical support for the parental selection for hybridization, tracking exogenous gene introgression and the construction of core germplasm collections [15]. In addition, karyotype analysis can be combined with genomic sequencing data to reveal the relationship between chromosomal structural variations and species differentiation. For instance, in the study of plants in the *Lauraceae* family, through chromosome-level genome assembly and karyotype analysis, the phenomenon of the incomplete lineage sorting of *Magnoliidae* during the differentiation of monocotyledons and dicotyledons was revealed [16].

In genetic research, karyotype analysis also offers significant support for gene mapping and functional studies. Take wheat research as an example—scholars have successfully identified gene loci associated with disease resistance and yield through karyotype analysis combined with gene mapping, thereby furnishing a theoretical foundation for crop breeding [17,18]. Moreover, karyotype analysis can also reveal the relationship between chromosomal structural variations and species adaptability [19,20]. For instance, in the study of plants in the *Asteraceae* family, karyotype analysis has found that chromosomal translocation events play an important role in species differentiation, offering a new perspective for understanding species adaptability and evolutionary mechanisms [21]. In conclusion, as an important method in plant taxonomy and genetic research, karyotype analysis can not only reveal the origin and evolutionary history of species but also provide important evidence for resolving taxonomic disputes and guiding breeding practices [22,23,24]. With the development of molecular biology techniques, karyotype analysis will be combined with other genomics methods to further advance research progress in plant taxonomy and genetics [9,25,26] to obtain better chromosome preparation results of winter turnip rape and determine the karyotype formula and the degree of asymmetry of winter turnip rape.

In the present study, we utilized the strong cold-tolerant winter turnip rape Longyou 7 to enhance staining efficacy and ascertain the karyotype formula and asymmetry. We first optimized several critical conditions for chromosome preparation, followed by karyotype analysis under optimal conditions, thereby establishing a theoretical foundation for further investigation into the phylogenetic evolution and genetic trends of this species.

## 2. Results

### 2.1. Optimization of Chromosome Preparation Techniques

#### 2.1.1. Determine the Best Time to Take Materials

To determine the optimal sampling time for the root tips of winter turnip rape, sampling was carried out at different time periods of the day. The results are shown in Table 1 and Supplementary S1. The optimal sampling time for the root tips of winter turnip rape is from 8:00 to 10:00 in the morning. During this period, the root tips of winter turnip rape grow more vigorously, and there are relatively more cells in metaphase in cell division, which is convenient for sampling and slide preparation.

#### 2.1.2. Effect of Different Pretreatment Times on Chromosomes

**Figure 1:**
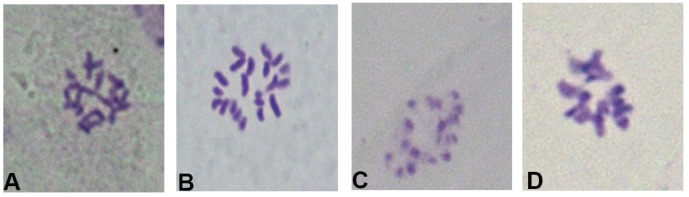
Chromosomes of winter turnip rape under different pretreatment times. (**A**) The chromo some map of winter turnip rape after 16 h of pretreatment. (**B**) The chromosome map of winter turnip rape after 20 h of pretreatment. (**C**) The chromosome map of winter turnip rape after 24 h of pretreatment. (**D**) The chromosome map of winter turnip rape after 48 h of pretreatment.

The primary objective of pretreatment was to obstruct cell division during metaphase by inhibiting and dismantling spindle filament formation, thereby increasing the number of cells in metaphase. Additionally, pretreatment alters cytoplasmic viscosity, promotes chromosome dispersion and contraction, and aids in compression and observation. Commonly used pretreatment methods include a composite method that combines physical, chemical, and mixed treatment [27]. Varied pretreatment durations significantly influenced the staining effect of winter turnip rape. Following 16 h of treatment with a mixture of cold water at 4 °C, the level of chromosome consolidation was markedly inadequate, exhibiting significant tailing phenomena, with chromosomes clustering together and demonstrating poor dispersion (Figure 1A); however, after 24 h and 48 h of the 4 °C ice–water mixture, it was found that some chromosomes were over-contracted and punctate and there was aggregation, which was no longer suitable for karyotyping (Figure 1C,D). The ideal pretreatment method for 20 h was identified due to its superior contractility and morphology (Figure 1B). Consequently, a duration of 20 h at a low temperature of 4 °C constituted the optimal pretreatment period.

#### 2.1.3. Effect of Fixation Time on Chromosome Preparation Efficiency

**Figure 2:**
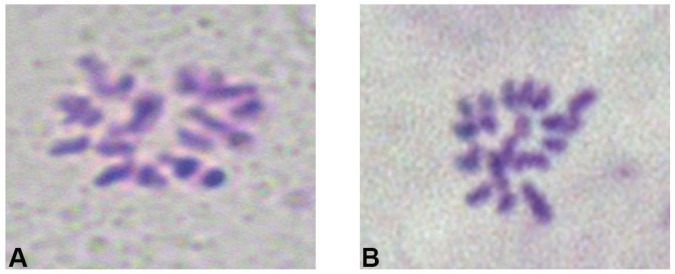
Chromosomes of winter turnip rape under different fixation times. (**A**) Chromosome map of winter turnip rape after 12 h fixation treatment. (**B**) Chromosome map of winter turnip rape after 24 h fixation treatment.

The purpose of fixation is to rapidly kill the material with a fixative that has strong penetrating power, precipitate the proteins, and preserve the original state of the material as much as possible. The processing time of Carnot’s solution has a significant impact on the preparation. Inadequate fixation duration will result in chromosome degradation during preparation, preventing the acquisition of intact chromosome morphology. Xiao et al. revealed that clear chromosome images of *Althaea rosea* root tips can be obtained after 24 h treatment [28]. In this experiment, the best results were achieved when using Carnoy’s fixative solution I (V absolute ethanol/V glacial acetic acid = 3:1) for 12 h. After 12 h fixation, the chromosomes had intact structures and clear shapes and numbers (Figure 2A). However, a 24 h fixation resulted in unclear chromosome structures, which was not conducive to observation (Figure 2B). In this experiment, Carnot’s fixative solution I (V absolute ethanol/V glacial acetic acid = 3:1) was used for 12 h, with intact structure and clear shape number (Figure 2A). However, fixation for 24 h resulted in ambiguous chromosome shape, which was not conducive to observation (Figure 2B).

#### 2.1.4. Effect of Different Dissociation Methods and Times on Chromosome Preparation Efficiency

The plant cells possess a cell wall, and the intercellular layer comprises pectin, complicating the extraction of the sheet to attain the intended result. Compression can proceed smoothly only after dissociation, the removal of the pectin layer between the cells, and the softening of the cell wall [29,30]. The acidolysis method generally uses 1 mol/L HCL to dissociate in a water bath at 60 °C for a few minutes to ten minutes or longer. The removal of the cell wall through a mixture of cellulase (1–5%) and pectinase (1–3%) is influenced by various factors, including enzyme concentration, quality, pH, duration, and temperature of the enzymatic hydrolysis. Typically, the enzymatic hydrolysis duration ranges from 2 to 5 h at room temperature.

**Figure 3:**
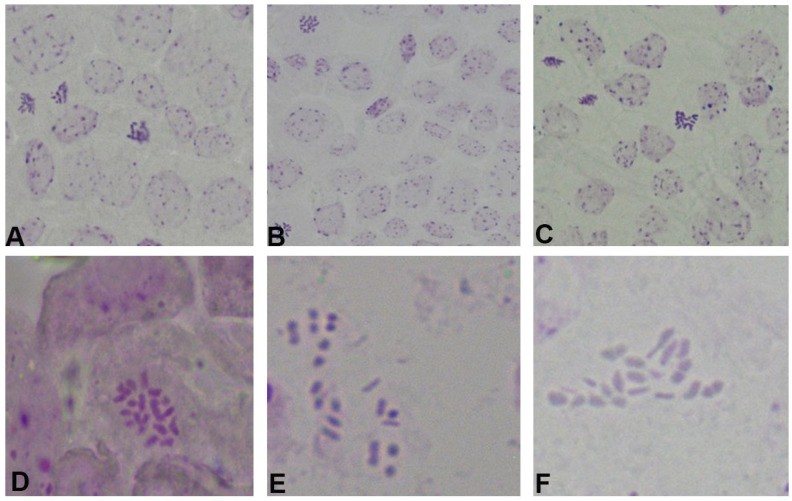
The effect of acid dissociation time and enzymolysis time on chromosomes. (**A**–**C**) The effects of acid digestion method with dissociation for 5 min, 10 min, and 15 min, respectively, on chromosomes. (**D**–**F**) The effects of enzymatic digestion with dissociation durations of 0.5 h, 1 h, and 1.5 h on chromosomes, respectively.

Acidolysis and enzymatic hydrolysis techniques yield superior metaphase division images of chromosomes, with comparison investigations indicating that treatment with 1 mol/L HCl for 10 to 15 min during acidolysis produces optimal results (Figure 3A–C). During enzymatic hydrolysis at 0.5 h, a significant quantity of cell walls remained intact, with cells accumulating and being insufficiently dispersed (Figure 3D). After 1 h of treatment, the enzymatic hydrolysis of the cell wall was deemed appropriate, resulting in the dissolution of the cell wall and dispersion of the cytoplasm, with chromosomes being relatively well distributed, facilitating counting (Figure 3E). However, at 1.5 h, cell rupture occurred and chromosomes were absent, indicating excessive enzymatic hydrolysis (Figure 3F). With the enzymatic hydrolysis time of 1 h and the enzymatic hydrolysis temperature of 37 °C, by both methods of enzymatic digestion and acidolysis, we obtain a clear number of chromosome images, but acidolysis is simpler and more effective.

### 2.2. Karyotype Analysis of Winter Turnip Rape Chromosomes

#### 2.2.1. Number of Chromosomes in Rapeseed

**Figure 4:**
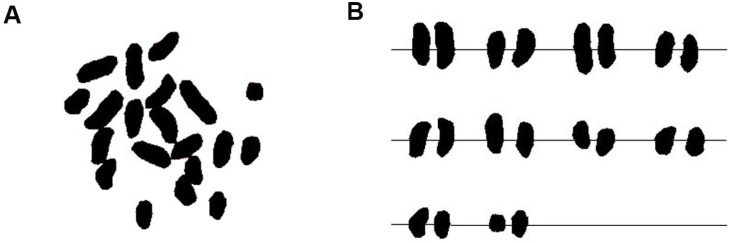
Chromosome morphology diagram and karyotype diagram of winter turnip rape. (**A**) Chromosome morphology diagram of winter turnip rape. (**B**) Karyotype diagram of chromosomes of winter turnip rape.

According to the standards established by Li Maoxue et al., the laboratory cell count must exceed 30. If over 85% of the cells exhibit a uniform and stable chromosome count, this can be regarded as the chromosome number in the plant [26]. In present study, 40 well-dispersed metaphase chromosome cells were selected for observation, and 36 cells were found to have 20 chromosomes (Figure 4), accounting for 90% of the counted cells. Therefore, the number of somatic chromosomes in winter turnip rape was determined to be 2n = 20.

#### 2.2.2. Karyotype Analysis

**Figure 5:**
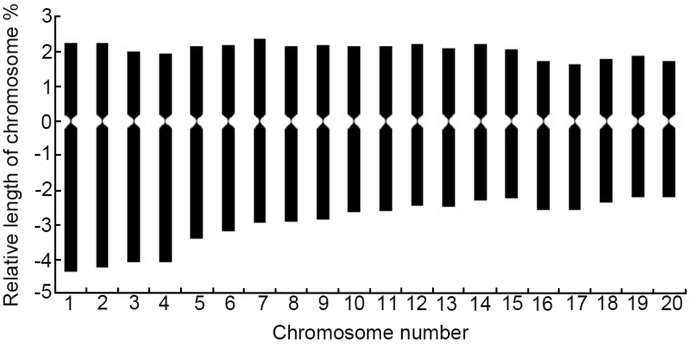
Karyotype pattern diagram of chromosomes of winter turnip rape.

Five well-dispersed and clear metaphase phases were selected for karyotyping. The winter turnip rape is diploid with a karyotype formula of K(2n) = 20 = 16m + 4sm, with central and near-middle centromeric chromosomes and no entourage (Table 2, Figure 5). The relative length of chromosomes ranged from 3.94~6.61, the coefficient of relative length of chromosomes ranged from 0.79~1.32, and the relative length of chromosomes was 2L + 6M_2_ + 12M_1_, among which L, M_2_, and M_1_ represented large chromosomes, middle chromosomes 2, and middle chromosomes 1, respectively. The arm ratio varied in the range of 1.05~2.11, the karyotype asymmetry coefficient was 58.85%, and the karyotype type belonged to type 2A (Supplementary S2 and S3).

## 3. Discussion

### 3.1. Staining and Slicing Technology

The technique of plant chromosome slide preparation is an experimental methodology encompassing disciplines such as plant genetics, chromosome engineering, plant cytotaxonomy, and plant cell biology [31,32]. The root tip is generally the most commonly used material for chromosome preparation in higher plants—because of its vigorous cell division, growth is not easily restricted by the season and it is easy to obtain [33,34]. Therefore, the plant material used in this study was the root tip of winter turnip rape. The chromosomes of winter turnip rape are smaller, and according to the electric field theory of the chromosomes of the Lima-de-Faria, the chromosomes of winter turnip rape belong to the microchromosomes [35]. When analyzing medium-sized chromosomes, even if there is an overlap of individual chromosomes, the outline of each chromosome can still be distinguished and it can generally be used for the karyotyping of chromosomes. But when chromosomes are tiny chromosomes, the overlap of some chromosomes can cause the outlines of the chromosomes to be indistinguishable or even impossible to count. This determines that the karyotype analysis of cabbage-type winter rape has higher requirements for the degree of chromosome dispersion and also has higher requirements for production technology. Like the chromosomes of winter turnip rape, the chromosomes of Plumbago auriculata are also microchromosomes. The average length of the entire set of its chromosomes is 0.40 μm [36]. This study builds upon their method and designs different sampling times, pretreatment durations, fixation times, and digestion times to compare and screen for the optimal chromosome preparation technique.

Most researchers will study the primary root tips of plants when performing a karyotype analysis of plants [37,38]. By collecting the root tips of winter turnip rape at different times, it was found that the cell division was most vigorous between 8:00 and 10:00 in a day, which was different from the 11:00–11:30 time of Pingyi sweet tea [39]. The best time for *Brassica alboglabra* var. *lutea* was 9:00 a.m. [40], and the time of collection was basically the same as that of this experiment. The degree of cell division is related to the preparation of the last chromosome, and the more cells in metaphase, the more favorable the karyotype analysis of chromosomes, so the time of sampling should be optimized.

Pretreatment is a crucial step in chromosome preparation technology, which not only enables the material to obtain more metaphase-splitting phases but also affects the degree of chromosome dispersion and clarity, so it is directly related to the chromosome preparation effect [32]. Sun et al. found that the morphology of chromosomes treated with colchicine for 6 h was better, while when treated with 8-hydroxyquinoline and saturated p-dichlorobenzene, the chromosomes were highly concentrated; when treated with ice water, the cross-overlap between chromosomes and adhesions were not dispersed [41]. However, the improved tablemaking technique used in the study of Tu Hongyan et al. eliminated the pretreatment step, and they directly carried out material extraction, acid dissociation, hypotonosis, staining, and tableting, which can also obtain good chromosomes with a clear shape and number [42]. These are the shortcomings of this experiment, which only optimized the pretreatment time of the ice–water mixture, and the optimal pretreatment time for winter turnip rape was 20 h at 4 °C. In conclusion, the optimization of pretreatment in this experiment can be further studied, such as the use of colchicine, a saturated aqueous solution of p-dichlorobenzene, etc.

The fixation time is generally set to 2~24 h; the time can be appropriately shortened if the material is small appropriately extended if the material is large, and the fixation effect is better for most plants under the condition of 4 °C using Carnot’s fixative solution I [43]. The Carnot’s fixative solution used in this study is a mixture of absolute ethanol and glacial acetic acid in a ratio of 3:1, and the effect of fixation time on the effect of chromosome preparation was compared. For root tip cells of winter turnip rape, fixing with Carnoy’s solution for 12 h can obtain good chromosome images. This is different from the study of *Alcea rosea* by Xiao Suxin et al., which may be related to the size of chromosomes [28]. Dissociation is also an important factor affecting the preparation effect of chromosomes. The purpose of dissociation is to break down and soften the cell wall so that cells can be easily separated, and at the same time, part of the cytoplasm can be removed so that the background of the cytoplasm is transparent, which is convenient for chromosome observation. In the preparation of chromosomes of winter turnip rape, acidolysis and enzymatic hydrolysis can obtain relatively good preparation results. In this test, 1 mol/L HCl treatment was used for 10~15 min, and the test found that the dissociation time was too short, which would lead to an insufficient dispersion of cells during tableting, and the coloring contrast of chromosomes and the cytoplasm of cells was too low, which was not easy to observe. However, if the dissociation time is too long, staining is more difficult, which is consistent with previous studies [27]. The results showed that the chromosomes were dispersed and morphologically clear after 20 h of pretreatment in a 4 °C ice–water mixture treatment. The structure of the chromosomes was obvious after 12 h treatment with fixation in Carnot’s fixative solution, and the dispersion degree of chromosomes was relatively good and the subsequent staining effect was good after 10~15 min treatment with 1 mol/L HCl. Finally, a Carbao magenta staining solution was used for 15 min and observed with a 100× oil immersion objective lens. A relatively good metaphase split phase can be obtained.

### 3.2. Chromosome Karyotype Analysis

Yang et al. believe that karyotype parameters obtained from karyotype analysis experiments can be used to study the growth and development of plants, compare the genetic relationships between different species of plants, and explain the evolutionary process of plants, which is an important basic data for understanding and studying plants [44,45]. In this study, the diploid chromosome number (2n = 20) of winter turnip rape is significantly lower than that of most cultivated species in *Brassicaceae* (e.g., *Brassica napus* 2n = 38, *Brassica juncea* 2n = 36), the latter of which are mostly formed by interspecific hybridization and polyploidization. Low ploidy and fewer chromosomes are generally considered characteristics of primitive groups, as they have not undergone complex chromosome doubling or structural rearrangements. Compared with model plants such as *Arabidopsis*, although winter turnip rape has more chromosomes, it belongs to the basic chromosome set (AA genome) in the genus *Brassica* without participating in allopolyploidization events, thus retaining the chromosome base number of ancestral species [46,47,48]. In the karyotype, 16 metacentric chromosomes (m type) account for 80%, and only 4 are submetacentric (sm type). The arm ratio is concentrated at 1.05–2.11, and the relative length variation range is small (3.94–6.61). This high proportion of metacentric chromosomes and uniform arm ratio distribution conform to the evolutionary theory of symmetric karyotypes being more primitive [49,50,51].

The karyotype of winter turnip rape cultivar Longyou 7 belongs to type 2A. According to Stebbins’ karyotype classification system, type 2A is a relatively symmetric karyotype commonly found in less evolved groups, while evolved groups mostly exhibit asymmetric karyotypes such as types 2B and 3A (for example, rice predominantly has the type 2B karyotype with an asymmetry coefficient of approximately 65%) [52,53,54,55]. Additionally, the karyotype asymmetry coefficient serves as an indicator to measure the evolutionary degree of organisms. Generally, the closer the coefficient is to 50%, the higher the symmetry, indicating a more primitive evolutionary status. The karyotype asymmetry coefficient of winter turnip rape (58.84%) is significantly lower than that of most cultivated crops [56]. For instance, *Brassica napus* has an asymmetry coefficient of about 62–65%, and wheat (2n = 42) can exceed 68%. This coefficient reflects the difference in chromosome length and the degree of arm ratio unevenness. A lower value indicates a more symmetric karyotype and a more primitive evolutionary position [57,58].

## 4. Materials and Methods

The tested rapeseed variety, “Longyou 7”, a strong cold-tolerant winter rapeseed variety (tolerating −32 °C, overwintering survival rate over 90%), was provided by the Department of Crop Genetics and Breeding, Gansu Agricultural University. Longyou 7 was developed by crossing Chenjiazui Oilseed Rape, a maternal parent with strong cold resistance, and Tianyou 1, a paternal parent with excellent yield traits. The hybrid combination was subjected to three rounds of recurrent selection under natural conditions in the F_2_ generation and subsequent generations. The healthy and well-nourished winter turnip rape seeds were disinfected with 10% sodium hypochlorite for 20 min, rinsed with sterile water 4~6 times, and then put into a Petri dish for germination at a temperature of 25 °C which was maintained in the dark until the root tip grew to 1–1.5 cm. The optimization of the chromosome, preparation, and karyotype analysis test were carried out. The experiment was carried out in the Key Laboratory of Characteristic Resource Utilization of the Hexi corridor in Gansu province.

**Figure 6:**
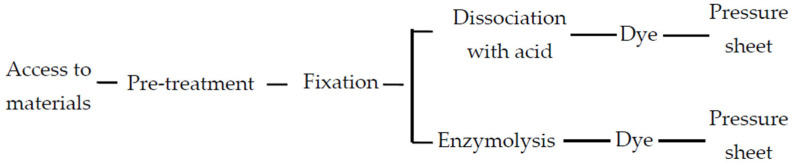
Improved process of pressure sheet.

In the present study, an improved compression method was used [59], and the operation process is shown in Figure 6. Using the root tip of winter turnip rape as the material, the design was optimized using four steps, including the collection time, pretreatment, fixation, and dissociation.

### 4.1. Optimization of Material Collection Time

The growth of plant meristems exhibits a certain periodicity. It is generally believed that the most appropriate time for sampling is during metaphase in cell division, typically between 08:00 and 11:00 or 13:00 and 15:00 [60]. However, studies have found that the optimal sampling time lacks good stability, and the proportion of metaphase chromosomes obtained even at the optimal time fluctuates significantly [61]. Some scholars have also pointed out that the cell cycle of different plants can vary under different temperature conditions, and using a fixed concept of circadian rhythm to understand the variable cell cycle is theoretically unfounded and cannot be confirmed in practice. Therefore, as long as the plant meristems are in a good active state, sampling can be performed at any time of the day [62]. Within the photoperiod from 8:00 to 18:00, sampling nodes were set at 2 h intervals, including a total of 5 sampling time points (i.e., 8:00, 10:00, 12:00, 14:00, 16:00). During each sampling, Longyou 7 seedlings with consistent growth status were randomly selected. Samples were quickly excised at 1–1.5 cm from the root tip (retaining the meristematic zone) using a sharp blade and immediately immersed in pre-cooled Carnot’s fixative (methanol: glacial acetic acid = 3:1) (Gansu Shenghuawei Trading Co., Ltd, Lanzhou, China).

### 4.2. Optimization of Preprocessing Time

The purpose of pretreatment is to inhibit or disrupt spindle formation, thereby arresting mitosis at the metaphase stage. It also induces a high condensation of chromosomes, shortening them to facilitate dispersion. Different pretreatment methods yield distinct effects. For instance, chemical solutions (e.g., mixtures of colchicine and 8-hydroxyquinoline), even at low concentrations, exert toxic effects on cells with intensity varying by temperature. In comparison, low-temperature pretreatment using an ice–water mixture is relatively safer [63]. In the present study, the material was prepared with a mixture of ice water and ice at a low temperature of 4 °C. The ice–water combination was set to 4 °C for 16 h, 20 h, 24 h, and 48 h, respectively, and each treatment was repeated in 5 sets to establish the ideal pretreatment condition time.

### 4.3. Optimization of Fixed Time

The purpose of fixation is to rapidly kill cells with a fixative, precipitating proteins while preserving their original structures as much as possible. Carnoy’s fixative I (anhydrous ethanol/glacial acetic acid = 3:1) is typically used for low-temperature fixation (approximately 4 °C). Although methanol may substitute anhydrous ethanol, its higher toxicity and stronger hardening effect on cell walls and chromosomes limit its application primarily to the wall-breaking hypotonic drying method [64]. This experiment employed Carnoy’s fixative I (V anhydrous ethanol/V glacial acetic acid = 3:1) at a low temperature (4 °C) for fixation. Two treatment durations (12 h and 24 h) were tested with five replicates per treatment to optimize fixation conditions.

### 4.4. Optimization of the Dissociation Stage

The core objective of dissociation is to disrupt the pectin layer and cell wall structure between cells, enabling cell dispersion for observing individual chromosome morphology under a microscope after squashing. Two dissociation methods were employed, namely the (1) acid digestion method using strong acid (1 mol/L HCl) at a high temperature (60 °C) to hydrolyze cellulose and pectin in the cell wall, rapidly breaking down cell adhesion. Dissociation times were set to 5 min, 10 min, and 15 min. (2) The enzymatic digestion method: At 25 °C, a specific catalytic action of pectinase (1%) and cellulase (2%) (Shanghai Yuanye Bio - Technology Co., Ltd, Shanghai, China) gently degrades cell wall components (pectin, cellulose) to maximize chromosome integrity. Dissociation times were set to 2 h, 3 h, and 4 h. Each treatment included 5 replicates to determine the optimal dissociation method and duration [13,61].

### 4.5. Staining and Microscopic Examination

After the root tip is dissociated, it was rinsed twice with distilled water, the root tip meristem part was cut and placed on a glass slide, and it was stained with a Carbol Fuchsin solution for 10~15 min. A microscopic examination was performed after conventional compression using a Leica microscope to observe, then 30 cells were selected with scattered chromosomes and good morphology to count the number of chromosomes. If more than 85% of the chromosome numbers are the same, the number of chromosomes can be determined [65]. Secondly, 5 cells with good division and clear chromosome morphology were selected and then directly photographed with a Leica camera (Leica DMI5000M, Wetzlar, Germany).

### 4.6. Karyotype Analysis

Metaphase cells with a clear number of 30 chromosomes were selected for chromosome counting, and karyotype analysis was performed using the criteria proposed by Li et al. to determine the location of the centromere. Chromosome karyotypes were paired and analyzed according to the chromosome size and morphological characteristics [13,65]. Relative chromosome length, arm ratio, and classification were calculated according to the nomenclature system established by Levan et al. [52]. The centromere index and relative length coefficient followed the standards proposed by Li and Chen [65]. Karyotype asymmetry coefficients were determined using the method described by ARANO [66], and karyotype types were classified based on Stebbins’ criteria [53,54].

## 5. Conclusions

This study optimized chromosome preparation protocols for root tip samples of winter turnip rape Longyou 7 and performed karyotype analysis. The optimal conditions were determined as follows: sampling time between 08:00 and 10:00; pretreatment with ice–water mixture at 4 °C for 20 h; fixation at 4 °C for 12 h; and hydrolysis in 1 mol/L HCl at 60 °C for 10–15 min. Karyotyping revealed a chromosome number of 2n = 20, with the karyotype formula 2n = 2x = 20 = 16m + 4sm. The karyotype asymmetry index was 58.85%, classifying the karyotype as the 2A type, which suggests a primitive evolutionary status. These findings provide a theoretical basis for further research on phylogenetic evolution and genetic inheritance patterns in the genus *Brassica*.

## Figures and Tables

**Table 1:** Effect of different collection times on metaphase of winter turnip rape.

Collection Time	Proportion of Metaphase Cell/%
8:00–10:00	8.57 ± 0.72
10:00–12:00	8.33 ± 2.75
12:00–14:00	8.46 ± 2.33
14:00–16:00	8.45 ± 2.96
16:00–18:00	8.26 ± 0.61

**Table 2:** Karyotypes of winter turnip rape.

Number of Chromosomes	Relative Length/%			Index of Relative Length	Type of Length	Arm Ratio (Long Arm/Short Arm)	Type of Chromosome
	Long Arm	Short Arm	Total Length				
1	4.36	2.25	6.61	1.32	L	1.94	sm
2	4.26	2.25	6.51	1.30	L	1.89	sm
3	4.08	2.01	6.09	1.22	M_2_	2.04	sm
4	4.08	1.94	6.02	1.20	M_2_	2.11	sm
5	3.41	2.15	5.56	1.11	M_2_	1.59	m
6	3.20	2.18	5.38	1.08	M_2_	1.47	m
7	2.96	2.36	5.32	1.06	M_2_	1.25	m
8	2.92	2.15	5.07	1.01	M_2_	1.36	m
9	2.85	2.18	5.03	1.01	M_1_	1.31	m
10	2.64	2.15	4.79	0.96	M_1_	1.23	m
11	2.60	2.15	4.75	0.95	M_1_	1.21	m
12	2.46	2.22	4.68	0.94	M_1_	1.11	m
13	2.50	2.11	4.61	0.92	M_1_	1.18	m
14	2.32	2.22	4.54	0.91	M_1_	1.05	m
15	2.25	2.08	4.33	0.87	M_1_	1.08	m
16	2.57	1.72	4.29	0.86	M_1_	1.49	m
17	2.57	1.65	4.22	0.85	M_1_	1.55	m
18	2.36	1.80	4.16	0.83	M_1_	1.31	m
19	2.22	1.87	4.09	0.82	M_1_	1.19	m
20	2.22	1.72	3.94	0.79	M_1_	1.29	m

## Data Availability

The original contributions presented in this study are included in the article. Further inquiries can be directed to the corresponding author.

## Supplementary Materials

The following supporting information can be downloaded at: https://www.mdpi.com/article/10.3390/ijms26157127/s1.
