# Supplementary material for: Optimization of Chromosome Preparation and Karyotype Analysis of Winter Turnip Rape (Brassica rape L.)
Source: Int J Mol Sci. 2025 Jul 24;26(15):7127. doi: 10.3390/ijms26157127 (PMC12346318; doi:10.3390/ijms26157127)

S1

	metaphase	total number	Proportion of Metaphas	sum	average
	20	251	0.079681		
	10	120	0.083333		
8：00-10：00	7	71	0.098592	0.428636	8.57%
	9	114	0.078947		
	17	193	0.088083		
	8	75	0.106667		
	13	157	0.082803		
10：00-12：00	17	296	0.057432	0.416364	8.33%
	9	158	0.056962		
	18	160	0.112500		
	2	39	0.051282		
	14	160	0.087500		
12：00-14：00	4	34	0.117647	0.423007	8.46%
	10	101	0.099010		
	5	74	0.067568		
	5	64	0.078125		
	2	36	0.055556		
14：00-16：00	10	72	0.138889	0.422343	8.45%
	4	65	0.061538		
	9	102	0.088235		
	8	91	0.087912		
	8	106	0.075472		
16：00-18：00	8	95	0.084211	0.412751	8.26%
	7	93	0.075269		
	8	89	0.089888		

8：00-10:00	10：00-12:00	12:00-14:00	14：00-16:00	16:00-18:00
0.079681275	0.106666667	0.051282051	0.078125	0.087912088
0.083333333	0.082802548	0.0875	0.055555556	0.075471698
0.098591549	0.057432432	0.117647059	0.138888889	0.084210526
0.078947368	0.056962025	0.099009901	0.061538462	0.075268817
0.088082902	0.1125	0.067567568	0.088235294	0.08988764

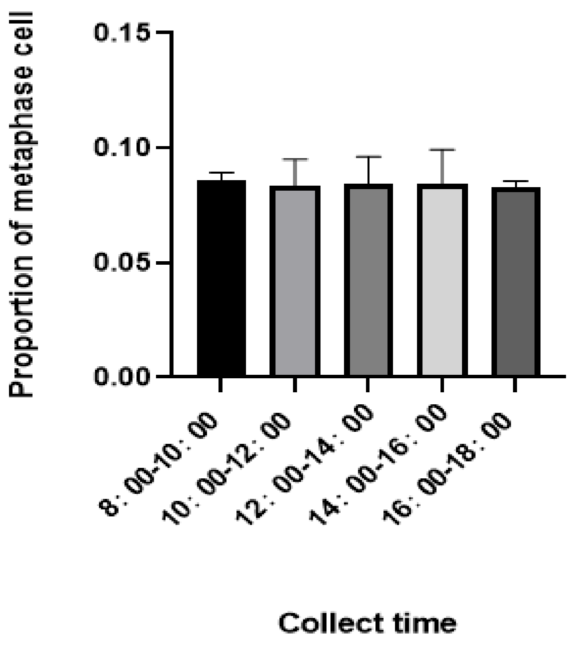

Tukey's multiple c	Mean Diff.	95.00% CI of diff.	Below threshold?	Summary	Adjusted P Value	
8：00-10：00 vs.	0.002455	-0.04048 to 0.04539	No	ns	0.9998	A-B
8：00-10：00 vs.	0.001126	-0.04181 to 0.04407	No	ns	>0.9999	A-C
8：00-10：00 vs.	0.001259	-0.04168 to 0.04420	No	ns	>0.9999	A-D
8：00-10：00 vs.	0.003177	-0.03976 to 0.04612	No	ns	0.9994	A-E
10：00-12：00 vs	-0.001329	-0.04427 to 0.04161	No	ns	>0.9999	B-C
10：00-12：00 vs	-0.001196	-0.04414 to 0.04174	No	ns	>0.9999	B-D
10：00-12：00 vs	0.0007223	-0.04222 to 0.04366	No	ns	>0.9999	B-E
12：00-14：00 vs	0.0001327	-0.04281 to 0.04307	No	ns	>0.9999	C-D
12：00-14：00 vs	0.002051	-0.04089 to 0.04499	No	ns	0.9999	C-E
14：00-16：00 vs	0.001918	-0.04102 to 0.04486	No	ns	>0.9999	D-E

Test details	Mean 1	Mean 2	Mean Diff.	SE of diff.	n1	n2	q	DF
8：00-10：00 vs.	0.08573	0.08327	0.002455	0.01435	5	5	0.2419	20
8：00-10：00 vs.	0.08573	0.0846	0.001126	0.01435	5	5	0.111	20
8：00-10：00 vs.	0.08573	0.08447	0.001259	0.01435	5	5	0.124	20
8：00-10：00 vs.	0.08573	0.08255	0.003177	0.01435	5	5	0.3131	20
10：00-12：00 vs	0.08327	0.0846	-0.001329	0.01435	5	5	0.1309	20
10：00-12：00 vs	0.08327	0.08447	-0.001196	0.01435	5	5	0.1179	20
10：00-12：00 vs	0.08327	0.08255	0.0007223	0.01435	5	5	0.07119	20
12：00-14：00 vs	0.0846	0.08447	0.0001327	0.01435	5	5	0.01308	20
12：00-14：00 vs	0.0846	0.08255	0.002051	0.01435	5	5	0.2021	20
14：00-16：00 vs	0.08447	0.08255	0.001918	0.01435	5	5		

S2

	Absolute length of chromosome（μm）						
Serial number	Long arm	Short arm	Total length	Relative length coefficient = chromosome length / average chromosome length	Length type	Arm ratio (Long arm/Short arm)	Chromosome type
	0.124	0.064	0.188	1.32	L	1.94	sm
	0.121	0.064	0.185	1.30	L	1.89	sm
	0.116	0.057	0.173	1.22	M2	2.04	sm
	0.116	0.055	0.171	1.20	M2	2.11	sm
	0.097	0.061	0.158	1.11	M2	1.59	m
	0.091	0.062	0.153	1.08	M2	1.47	m
	0.084	0.067	0.151	1.06	M2	1.25	m
	0.083	0.061	0.144	1.01	M2	1.36	m
	0.081	0.062	0.143	1.01	M1	1.31	m
	0.075	0.061	0.136	0.96	M1	1.23	m
	0.074	0.061	0.135	0.95	M1	1.21	m
	0.070	0.063	0.133	0.94	M1	1.11	m
	0.071	0.060	0.131	0.92	M1	1.18	m
	0.066	0.063	0.129	0.91	M1	1.05	m
	0.064	0.059	0.123	0.87	M1	1.08	m
	0.073	0.049	0.122	0.86	M1	1.49	m
	0.073	0.047	0.120	0.85	M1	1.55	m
	0.067	0.051	0.118	0.83	M1	1.31	m
	0.063	0.053	0.116	0.82	M1	1.19	m
	0.063	0.049	0.112	0.79	M1	1.29	m
sum	1.672	1.169	2.841				
average			0.142				

S3

Relative length of rapeseed chromosomes (%)

Serial number	Long arm	Short arm	Total length
1	-4.36	2.25	6.61
2	-4.26	2.25	6.51
3	-4.08	2.01	6.09
4	-4.08	1.94	6.02
5	-3.41	2.15	5.56
6	-3.20	2.18	5.38
7	-2.96	2.36	5.32
8	-2.92	2.15	5.07
9	-2.85	2.18	5.03
10	-2.64	2.15	4.79
11	-2.60	2.15	4.75
12	-2.46	2.22	4.68
13	-2.50	2.11	4.61
14	-2.32	2.22	4.54
15	-2.25	2.08	4.33
16	-2.57	1.72	4.29
17	-2.57	1.65	4.22
18	-2.36	1.80	4.16
19	-2.22	1.87	4.09
20	-2.22	1.72	3.94

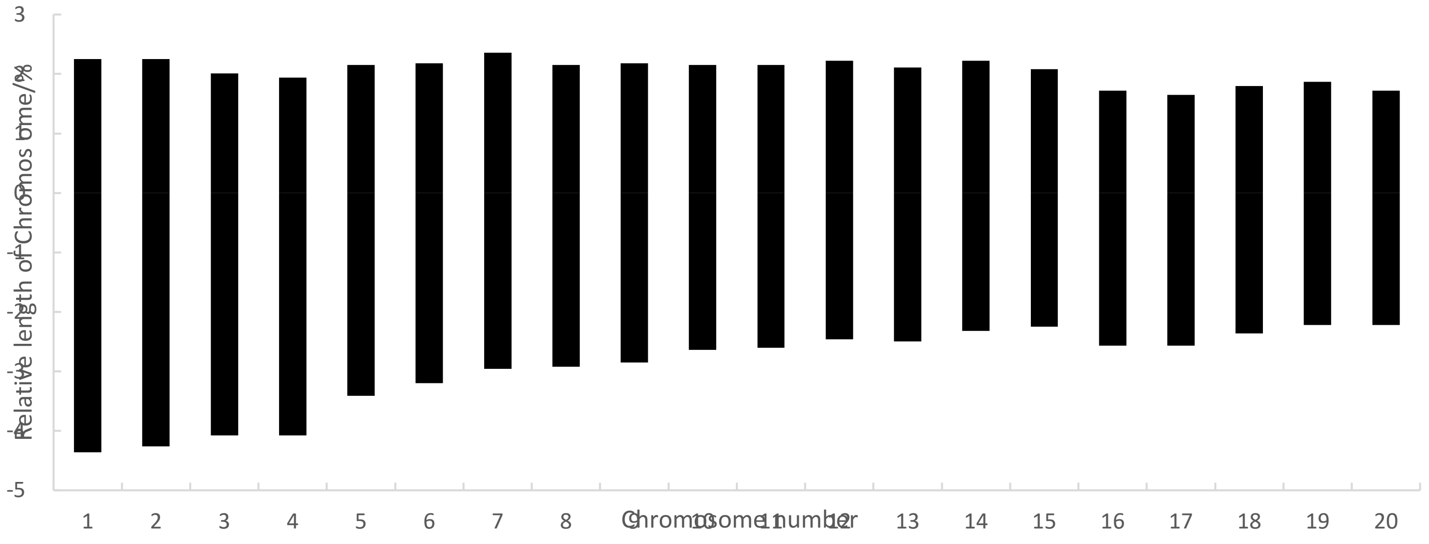

Supplement: Supplementary file 1 [file ijms-26-07127-s001.zip › ijms-3681617-supplementary.pdf]
